# Reduction of Coronavirus Burden With Mass Azithromycin Distribution

**DOI:** 10.1093/cid/ciaa606

**Published:** 2020-05-19

**Authors:** Thuy Doan, Armin Hinterwirth, Ahmed M Arzika, Lee Worden, Cindi Chen, Lina Zhong, Catherine E Oldenburg, Jeremy D Keenan, Thomas M Lietman

**Affiliations:** 1 Francis I. Proctor Foundation, San Francisco, California, USA; 2 University of California, San Francisco, San Francisco, California, USA; 3 Carter Center, Niamey, Niger

**Keywords:** azithromycin, coronavirus, children, virome, Niger

## Abstract

We evaluated the potential antiviral effects of azithromycin on the nasopharyngeal virome of Nigerien children who had received multiple rounds of mass drug administration. We found that the respiratory burden of non–severe acute respiratory syndrome coronaviruses was decreased with azithromycin distributions.

**Clinical Trials Registration**. NCT02047981.

Mass oral azithromycin distribution decreased childhood mortality in sub-Saharan Africa [1]. Verbal autopsy revealed fewer deaths in communities treated with azithromycin from a number of infectious diagnoses, including dysentery and pneumonia [2]. Consistent with azithromycin’s antibacterial properties, the gut pathogen load was reduced after the fourth biannual azithromycin distribution [3]. Because azithromycin may also have antiviral properties [4, 5], we performed an exploratory analysis on the effects of azithromycin on respiratory viral pathogens.

## METHODS

### Trial Oversight

We obtained ethical approval for the study from the University of California, San Francisco (UCSF) Committee for Human Research and the Ethical Committee of the Niger Ministry of Health (institutional review board number 10-01036). The study was undertaken in accordance with the Declaration of Helsinki. We obtained verbal informed consent from guardians of children prior to treatment and swab collection. No incentives were offered.

### Eligibility

As part of MORDOR (Macrolides Oraux pour Réduire les Décès avec un Oeil sur la Résistance) I and II (NCT02047981) [3], 30 communities in the Dosso region of Niger were randomly selected for enrollment in a smaller sister trial to assess for infectious disease outcomes. The randomization unit was at the community level. All children aged 1–59 months and weighing at least 3800 g were eligible for treatment. One community declined further participation after 24 months (Supplementary Figure 1). Therefore, there were 29 participating communities at the 36-month time point.

### Intervention

Children aged 1–59 months were randomized to receive 1 oral dose of placebo or azithromycin (height-based dosing to a target dose of ≥ 20 mg/kg) every 6 months for 3 years. All field and laboratory personnel were masked to the assignments.

### Sample Collection

Approximately 40 children from each of the 30 villages were randomly selected for sample collection at each time point (Supplementary Figure 1). The children selected for sample collection at baseline may not be the same children selected at subsequent time points, and some children aged out of eligibility during the trial. Nasopharyngeal swabs were obtained at baseline (prior to treatment), 24 months (6 months after the fourth treatment), and 36 months (6 months after the sixth treatment). Samples were immediately placed in DNA/RNA Shield (Zymo Research) to deactivate pathogens and preserve nucleic acid integrity. The samples were placed on ice packs in the field, stored at −20°C in Niger, and shipped to UCSF for long-term storage at −80°C until sample processing. The collection dates for the baseline, 24-month, and 36-month time points were 11 March–17 June 2015, 17 March–14 June 2017, and 11 May–1 July 2018, respectively.

### RNA Sequencing and Data Analysis

Ten samples from each village, at each time point, were randomly chosen (n = 890) for deep RNA sequencing to evaluate for both RNA and DNA viruses (Supplementary Figure 1 and Supplementary Table 1) [3]. Total RNA was extracted from the nasopharyngeal samples using the QIAGEN Allprep DNA/RNA Micro Kit per the manufacturer’s instructions. Sequencing libraries were prepared and sequenced as previously described [3]. In brief, 25 ng of total RNA was converted to double-stranded complementary DNA (cDNA). The cDNA was converted to Illumina sequencing libraries using the NEBNext Ultra II library preparation kit and amplified with 11 polymerase chain reaction cycles. Samples were sequenced on either the Illumina HiSeq 2500 or NovaSeq 6000 instrument using 125-base paired-end sequencing. Sequencing data were analyzed using a rapid, in-house computational pipeline to classify sequencing reads by comparison to the entire National Center for Biotechnology Information nucleotide reference database [3, 6]. DESeq2 was used to perform differential abundance analysis on the DNA and RNA viruses identified across all samples [7]. Topconfects algorithm was used to determine the confident effect sizes [8].

## RESULTS

**Figure 1.**
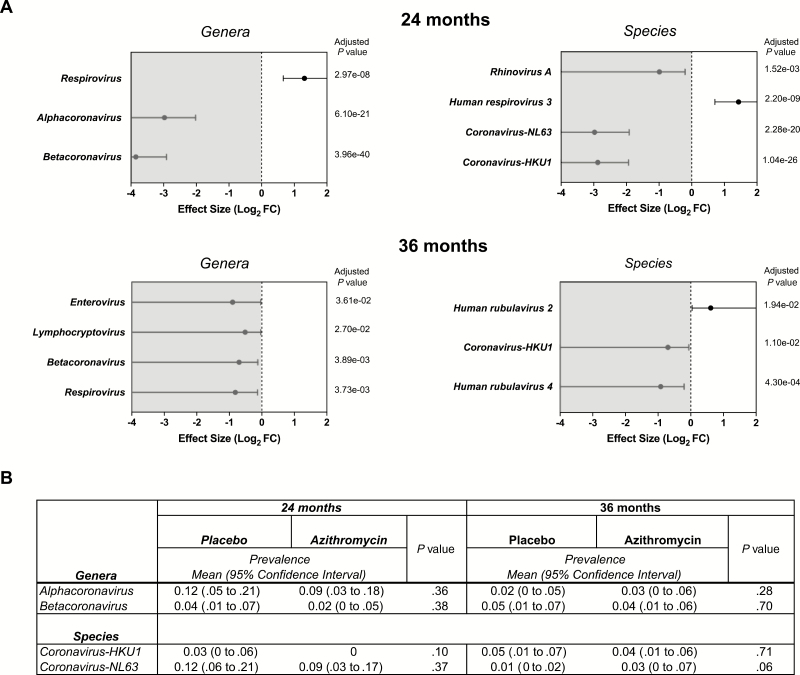
Relative abundance and prevalence of respiratory viruses between placebo- and azithromycin-treated children. *A*, Top virus genera or species at 5% false discovery rate at 24 and 36 months. For each genus or species, the dot shows the log_2_ fold change (FC) with confidence bound. Values in the nonshaded area represent more relative abundance in the azithromycin-treated group, whereas values in the shaded area represent more relative abundance in the placebo-treated group. Benjamini-Hochberg correction was used to determine the adjusted *P* values. *B*, *Coronavirus* prevalence at 24 and 36 months. Analysis of covariance *P* values is shown.

We analyzed 890 nasopharyngeal samples from preschool children in the Dosso region of Niger that were collected from 2015 to 2018. Characteristics of children whose samples were sequenced and analyzed are shown in Supplementary Table 1. At baseline, prior to treatment, *Alphacoronavirus* was more abundant in the nasopharynx of children randomized to the azithromycin arm (Supplementary Figure 2). There was no difference in either abundance or prevalence of other virus genera between arms. At 24 months, children from villages that underwent 4 azithromycin treatments had an 8-fold reduction in *Alphacoronavirus* and a 14-fold reduction in *Betacoronavirus* compared to children from villages treated with placebo (Figure 1A). At 36 months, *Betacoronavirus* was again observed to be differentially abundant between treatment groups, but with a modest 1.6-fold relative reduction in the azithromycin arm. Other genera found to be differential between treatments were *Enterovirus*, *Respirovirus*, and *Lymphocryptovirus*. At the species level, *Coronavirus-HKU1* and *Coronavirus-NL63* abundances were significantly reduced in the azithromycin group at 24 months, and again at 36 months (*Coronavirus-HKU1*) (Figure 1A). We were unable to detect a significant change in the proportion of children who harbored any evidence of coronavirus with azithromycin compared to placebo at either the 24- or 36-month time points (Figure 1B). These results suggest that mass azithromycin distribution may decrease viral load, but not the prevalence of colonization in a community.

## Discussion

In this study, children who received biannual azithromycin administrations had a relative reduction in non–severe acute respiratory syndrome (SARS) coronavirus load in the nasopharynx compared to children who received biannual placebo administrations.

While the viruses identified in this study all have the potential to cause diseases, the detection of *Coronavirus-HKU1* and *Coronavirus-NL63* is notable given the SARS epidemic in 2003 and the current pandemic with the novel coronavirus (SARS-CoV-2). These are enveloped single-stranded positive-sense RNA viruses. In particular, *Coronavirus-HKU1* belongs to the same genus as SARS-CoV-2. Both *Coronavirus-HKU1* and *Coronavirus-NL63* are human pathogens that have been reported worldwide [9, 10]. Disease manifestations include respiratory tract infections, meningitis, and gastroenteritis, and are generally self-limiting, although deaths have been documented in immunocompromised patients [11]. Whether mass azithromycin distribution provides herd effects or directly protects a treated child is unclear, as this study was not designed to determine the effects of mass azithromycin distribution on viral infections causing mortality. It is also unclear if clinical severity is dependent on viral load.

Macrolides have long been hypothesized to have therapeutic effects on viral infections, either via their anti-inflammatory effects or their off-target effects on viral replication. Recently, azithromycin has been shown to have activity against SARS-CoV-2 in vitro [12]. Preliminary evidence has also suggested that azithromycin may reduce viral load in patients with SARS-CoV-2 infection, although these studies were nonrandomized and suffered from small sample sizes [13]. Therefore, it remains to be determined if the observations seen in this study are translatable to SARS-CoV-2 or if treatment with azithromycin provides any clinical protection against SARS-CoV-2.

This cluster-randomized controlled trial is limited by the absence of data on respiratory symptoms and the nonprespecified nature of the analyses. Moreover, the generalizability of the results outside of preschool Nigerien children is unclear.

In summary, children in Niger treated with biannual mass azithromycin distributions experienced an associated decrease in the respiratory viral burden of non-SARS coronaviruses.

## Supplementary Data

Supplementary materials are available at *Clinical Infectious Diseases* online. Consisting of data provided by the authors to benefit the reader, the posted materials are not copyedited and are the sole responsibility of the authors, so questions or comments should be addressed to the corresponding author.

ciaa606_suppl_Supplementary_Material

## Notes

***Financial support.*** This work was funded by the Bill & Melinda Gates Foundation; the National Eye Institute of the National Institutes of Health (award number K08EY026986); the Peierls Foundation; a Research to Prevent Blindness Career Development Award; and an unrestricted grant from Research to Prevent Blindness. Pfizer donated all the medications used in this study.

***Potential conflicts of interest.*** The authors: No reported conflicts of interest. All authors have submitted the ICMJE Form for Disclosure of Potential Conflicts of Interest.
