# Supplementary material for: Reduction of Coronavirus Burden With Mass Azithromycin Distribution
Source: Clin Infect Dis. 2020 May 19;71(16):2282–4. doi: 10.1093/cid/ciaa606 (PMC7314118; doi:10.1093/cid/ciaa606)
Supplement: ciaa606_suppl_Supplementary_Material [file ciaa606_suppl_supplementary_material.docx]

**Table S1: Demographics of Analyzed Participants**

**Figure S1.** **Trial Profile**

**Figure S2: Relative abundance and prevalence of respiratory viruses between placebo and azithromycin treated children at baseline.** A. Top virus genera or species at 5% false discovery rate (FDR). For each genus or species, the dot shows the log_2_ fold change with confidence bound. Values in the non-shaded area represent more relative abundance in the azithromycin treated group whereas values in the shaded area represent more relative abundance in the placebo treated group. B. Virus prevalence at the genus level. Abbreviations: FC, fold change.
